# Drug‐related teratogenic and pathologic causes of birth defects in a tertiary hospital in Southwestern Nigeria

**DOI:** 10.1002/prp2.452

**Published:** 2019-02-06

**Authors:** Ifeanyichukwu Offor, Olufunsho Awodele, Kazeem A. Oshikoya

**Affiliations:** ^1^ Department of Pharmacology, Therapeutics & Toxicology College of Medicine University of Lagos Lagos Nigeria; ^2^ Department of Pharmacology College of Medicine Lagos State University Lagos Nigeria

**Keywords:** birth abnormalities, birth defects, congenital anomalies, newborns, teratogen, teratogenic

## Abstract

Birth defects are important causes of neonatal morbidity and mortality. A good understanding of the etiology is a vital step toward developing improved treatment and preventive strategies. We conducted an audit of medical records of newborns with birth abnormalities in a tertiary hospital over a 10‐year period, using a Pro forma designed to collect information on obstetric history, antenatal history, sociodemographics of parents, and the type of birth abnormality. Of the 180 medical records reviewed, female babies were 92 (51.1%) and male babies were 86 (47.8%). The mean age of the fathers was 38.2 + 6.2, and mothers 31.8 + 4.9. Majority 115 (63.9%) of the mothers had records of acute illnesses, and 23 (12.8%) chronic illnesses during pregnancy. Unspecified febrile illness 44 (38.3%), malaria 40 (34.8%), typhoid 8 (6.9%), hypertension 13 (56.5%), pregestational diabetes 4 (17.4%), and HIV 3 (13.0%) were the commonest maternal pathologies. Most of the documented birth abnormalities were Down's syndrome 34 (15.2%); congenital hydrocephalus 32 (14.3%); acyanotic congenital heart defect 30 (13.4%); deformity of the digits 26 (11.6%); and ventricular septal defect 20 (8.9%). The prevalence of maternal pathologies calls for concern, as these may be implicated in birth defects, therefore should be further investigated in future studies.

**Abbreviations**
ACHD: acyanotic congenital heart defect
CHD: congenital heart defect
LUTH: Lagos University Teaching Hospital
SVD: spontaneous vertex delivery
TT: tetanus toxoid
VSD: ventricular septal defect

## 1 INTRODUCTION

Congenital malformation otherwise known as birth defects or congenital abnormalities are structural, functional, genetic, or behavioral anomalies, including metabolic disorders that occur during fetal development and can be diagnosed prenatally, at birth or later in life.^1^, ^2^ They are important causes of adverse pregnancy outcomes, neonatal morbidity, and mortality in developed and developing countries.^3^

According to the US Center for Disease Control, major birth defects occur in one of every 33 births,^4^ and an estimated 7.9 million babies were affected worldwide in 2006.^5^ In Africa, neonatal and perinatal deaths due to congenital abnormalities are much higher compared to Europe. In Tanzania, a high perinatal death of 66.7% and neonatal death of 18.5% are caused by congenital malformations.^6^ In Nigeria, an earlier study reported about 16% of total perinatal deaths due to congenital malformation, with cardiovascular malformations accounting for 40% of the perinatal deaths.^7^ However, in Europe, a perinatal death of 2.0% in the form of stillbirths or fetal deaths and neonatal deaths (2.5%) in the first week of life are attributed to congenital anomaly.^8^ In Nigeria, available data on congenital malformations are mostly from hospital‐based studies.^9^, ^10^, ^11^ Abbey et al.^9^ in a descriptive retrospective cross‐sectional study conducted in a Nigerian teaching hospital reported a prevalence of 20.73 cases per 1000 live births, with the frequency in unbooked maternities significantly higher than the booked. Also, Obu et al.^11^ in a cross‐sectional retrospective review of medical records in a tertiary hospital in south‐eastern part of Nigeria reported a prevalence of 2.8%. Surgical birth defects including cleft lip/cleft palate and neural tube defects were the commonest birth defects. Similarly, Ekanem et al.^10^ in a review of birth registers reported a total of 452 cases of birth malformations of 127 929 recorded births.

About 50%‐60% of all congenital malformations have no specific cause, while others are associated with known causes or risk factors.^1^, ^2^ The known causes include maternal pathologies such as fever,^12^ pregestational diabetes,^13^ Zika virus infection,^14^ syphilis and rubella infection,^1^, ^2^ amongst others. Factors such as twining, genetic abnormalities, and family history of birth defect have also been implicated in various birth malformations.^13^, ^15^ Poor maternal nutritional status such as deficiencies in iodine and folic acid are linked to major congenital malformations like neural tube defect.^16^ Conversely, high intake of vitamin A and its derivatives during pregnancy may affect the normal development of an embryo or fetus.^17^ Maternal prescription drug use, including anticonvulsants, anticancer agents, and certain antidepressants may increase the risk of congenital malformations.^18^, ^19^ Advanced maternal and paternal ages are known risk factors for Down's syndrome.^20^, ^21^ Consanguinity increases the prevalence of rare genetic congenital abnormalities and nearly doubles the risk for neonatal and childhood death.^1^, ^2^ The use of social drugs such as alcohol and maternal exposure to tobacco smoke are also culpable factors of congenital malformation.^22^, ^23^ Finally, over 90% of severe congenital anomalies are found in low and middle‐income countries where pregnant women often lack access to adequate and quality food.^1^, ^2^

Some of the public health measures recommended by the WHO to decrease the frequency of certain birth defects include adequate dietary intake of vitamins and minerals, particularly folic acid in adolescent girls and mothers, preventing maternal exposure to harmful substances, adequate prenatal vaccination, rational prescribing in pregnancy, amongst others.^1^, ^2^

Understanding the etiology of birth abnormalities is a vital step toward developing improved treatment and preventive strategies of congenital defects.^1^, ^2^ To the best of our knowledge, no study has attempted to investigate the teratogenic and pathologic risk factors of birth malformations in a hospital‐based cohort in this part of the country. Therefore, the aim of this study was to decipher the teratogenic and pathologic risk factors of birth abnormalities by exploring the medical records of newborns with birth abnormalities over a 10‐year period (from 2006 to 2016) in a tertiary hospital located in southwestern Nigeria.

## 2 MATERIALS AND METHODS

The study center, Lagos University Teaching Hospital (LUTH) is a major tertiary hospital in Southwestern Nigeria, which often serves as a referral center for patients in Lagos State and its environs. Most of the cases of birth abnormalities in this hospital were referred from other hospitals. At the Medical Records Department, eligible cases were identified from the birth defect registry; the file numbers were used to recover the case files, and the cases were thoroughly reviewed one after the other by the investigator. Relevant information such as the demographics of the babies and their parents, antenatal and obstetric histories, parents’ social histories, specific diagnosis made with regard to birth abnormalities, types of birth abnormality, time birth abnormality was diagnosed, pathologic state of the parents, exposure to known teratogens during pregnancy, and pregnancy outcomes (whether live birth or stillbirth), were extracted using a well‐structured Pro forma.

### 2.1 Ethical approval

The ethical approval for this study was granted by the Health Research Ethics Committee of LUTH, Idi‐araba, Lagos, on April 21, 2017. Approval Number: ADM/DCST/HREC/APP/1564.

### 2.2 Statistical analysis

Relevant data were entered into Microsoft Excel spread sheet to check for accuracy. Thereafter, data were coded and loaded into Statistical Package for Social Sciences IBM SPSS, Version 20.0 (IBM Corp., Armonk, NY) for analysis. Descriptive statistics on sample characteristics such as frequency and percentages were computed. Chi‐square test was used to determine if important covariates such as booking status, medical condition in pregnancy, acute illness in pregnancy, self‐medication, or prescription drugs use during pregnancy have statistically significant association with the number of birth abnormalities.

## 3 RESULTS

### 3.1 Obstetric history of the newborns

The full obstetric history of the babies is presented in Table 1. A total of 186 cases with diagnosis of congenital anomalies were identified from the birth defect registry from January 2006 to December 2016. However, only 180 case notes were available for data abstraction. Amongst these, 10 (5.6%) were delivered in LUTH while majority 159 (88.3%) of the cases were referred from other centers including private hospitals, general hospitals, health centers, amongst others. The place of booking or delivery was not specified in 11 (6.1%) of the cases. Female babies were more in number 92 (51.1%), while the male babies were 86 (47.8%). Baby's sex was not specified in two (1.1%) of the cases. Majority 138 (76.7%) of the babies were delivered at term, whereas, preterm babies were only seven (3.9%). Gestational age at delivery was not specified in 35 (19.4%) of the cases. Most of the babies 137 (76.1%) were delivered through spontaneous vertex delivery (SVD). Babies delivered via elective and emergency cesarean sections were 24 (13.3%) and 9 (5.0%), respectively. Mode of delivery was not specified in eight (4.4%) of the cases. Most of the babies 138 (76.7%) were products of natural conception, only two (1.1%) were conceived via IVF. The mode of conception was not specified in 40 (22.2%) of the cases. Only three (1.7%) of the cases were twin while 170 (94.4%) were single birth. The number of delivery was not specified in seven (3.9%) of the cases. APGAR scores were not documented in almost all 170 (94.4%) cases. Majority 133 (73.9%) of the babies did not have birth asphyxia, while history of birth asphyxia was not documented in 33 (18.3%) of the cases. Only a few 14 (7.8%) of the babies had history of birth asphyxia. Most of the babies 103 (57.2%) had birth weight ≥2.50 kg, whereas, 44 (24.4%) had birth weight ≤2.49 kg. Birth weight was not specified in 33 (18.3%) of the cases. In terms of birth order in the family, most of the babies 54 (30.0%) were second born in the family, 48 (26.7%) firstborn, 32 (17.8%) third, and 19 (10.6%) fourth born babies. Majority 113 (62.8%) of the cases of birth abnormalities were diagnosed postpartum while 61 (33.9%) were diagnosed during postnatal follow‐up.

**Table 1:** Obstetric history of the babies

Variable	Frequency	Percentage
Booking status		
Booked	10	5.6
Unbooked	159	88.3
Not specified	11	6.1
Total	180	100.0
Gender		
Male	86	47.8
Female	92	51.1
Not specified	2	1.1
Total	180	100.0
Gestational age		
Preterm	7	3.9
Term	138	76.7
Not specified	35	19.4
Total	180	100.0
Product of IVF		
Yes	2	1.1
No	138	76.7
Not specified	40	22.2
Total	180	100.0
Mode of delivery		
Spontaneous vertex delivery	137	76.1
Elective cesarean section	24	13.3
Emergency cesarean section	9	5.0
Assisted delivery	2	1.1
Not specified	8	4.4
Total	180	100.0
Number of delivery		
One	170	94.4
Two	3	1.7
Not specified	7	3.9
Total	180	100.0
Birth weight (kg)		
≤2.49	44	24.4
≥2.50	103	57.2
Not specified	33	18.3
Total	180	100.0
Birth order in the family		
First	48	26.7
Second	54	30.0
Third	32	17.8
Fourth	19	10.6
Fifth	6	3.3
Sixth	2	1.1
Seven	1	0.6
Not specified	18	10.0
Total	180	100.0
APGAR (1 min)		
Three	2	1.1
Four	1	0.6
Five	1	0.6
Six	1	0.6
Seven	2	1.1
Eight	1	0.6
Nine	1	0.6
Ten	1	0.6
Not specified	170	94.4
Total	180	100.0
APGAR (5 min)		
Three	2	1.1
Seven	1	0.6
Eight	1	0.6
Nine	2	1.1
Ten	4	2.2
Not specified	170	94.4
Total	180	100.0
Time anomaly was diagnosed		
Intrapartum	5	2.8
Postpartum	113	62.8
Prenatal	1	0.6
Postnatal follow‐up	61	33.9
Total	180	100.0
Birth asphyxia		
Yes	14	7.8
No	133	73.9
Not specified	33	18.3
Total	180	100.0
Severity of asphyxia		
Mild	2	14.3
Moderate	5	35.7
Severe	1	7.1
Not specified	6	42.8
Total	14	100.0

### 3.2 Sociodemographics of the parents

The sociodemographics of the parents are presented in Tables 2 and 3. The mean age of the mothers was 31.8 ± 4.9. A majority 70 (38.9%) of the mothers were 33‐38 years old, 65 (36.6%) 26‐32 years, 21 (11.7%) 19‐25 years, and 9 (5.0%) 39‐43 years old. Maternal age was not specified in 15 (8.3%) of the cases. Majority 150 (83.3%) of the mothers were in a monogamous and nonconsanguineous 158 (87.8%) marriages. The mothers were mostly into the business of trading 56 (31.1%). However, 35 (19.4%) were full‐time house wives, 28 (15.6%) were working class professionals, and 22 (12.2%) were artisans. Maternal occupation was not specified in 21 (11.7%) of the cases. A majority of the mothers were nonsmokers 157 (87.2%), nonalcoholics 152 (84.4%), and do not use social drugs 157 (87.2%). There was no history of folic acid use prior to conception in most 147 (81.7%) of the mothers.

**Table 2:** Sociodemographic of the mothers

Variable	Frequency	Percentage
Use of other social drugs		
No	157	87.2
Not specified	23	12.8
Total	180	100.0
Previous stillbirth		
No	145	80.6
Not specified	35	19.4
Total	180	100.0
Preconceptional folic acid use		
No	147	81.7
Not specified	33	18.3
Total	180	100.0
Past IVF use		
No	139	77.2
Not Specified	41	22.8
Total	180	100.0

**Table 3:** Sociodemographic of the fathers

Variable	Frequency	Percentage
Age of father (years)		
21‐30	17	9.4
31‐40	97	53.9
41‐50	41	22.8
51‐60	5	2.8
Not specified	20	11.1
Total	180	100.0
Occupation		
Professional	45	25.0
Artisan	38	21.1
Business/trading	40	22.2
Others	36	20.0
Not specified	21	11.7
Total	180	100.0
Level of education		
Primary	11	6.1
Secondary	74	41.1
Tertiary	70	38.9
Not specified	25	13.9
Total	180	100.0
Smokes cigarette		
Yes	9	5.0
No	151	83.9
Not specified	20	11.1
Total	180	100.0
Alcohol intake		
Yes	30	16.7
No	132	73.3
Not specified	18	10.0
Total	180	100.0
Use of social drugs		
Yes	2	1.1
No	157	87.2
Not specified	21	11.7
Total	180	100.0

The mean age of the fathers was 38.2 ± 6.2. Most of the fathers 97 (53.9%) were 31‐40 years old. Middle aged fathers (41‐50 years) were 41 (22.8%), while 17 (9.4%) of the fathers were between 21 and 30 years old. Paternal age was not documented in 20 (11.1%) of the cases. Unlike the mothers, most of the fathers were professionals 45 (25.0%), artisans and traders were 38 (21.1%), and 40 (22.2%), respectively. Paternal occupations were not documented in 21 (11.7%) of the cases. Just like the mothers, most of the fathers were nonsmokers 151 (83.9%), nonalcoholics 132 (73.3%), and do not use social drugs 157 (87.2%).

### 3.3 Antenatal history

The full maternal antenatal history are presented in Table 4. Amongst the unbooked cases, 90 (50.0%) received antenatal in private hospitals, general hospitals 50 (27.8%), health centers six (3.3%), traditional birth attendant five (2.8%), and nurses shop three (1.7%). The number of women who received antenatal care in the church, maternity home, and referral from another teaching hospital were one (0.6%) each. Place of antenatal was not specified in 24 (13.3%) cases. Most of the cases 152 (84.4%) had no documented family history of congenital anomalies. Also, most 148 (79.4%) of the cases had no documented history of maternal herbal drug use. Also, majority 146 (81.1%) had no history of maternal self‐medication during pregnancy. However, a majority 115 (63.9%) of the mothers had acute illnesses during pregnancy. The acute illnesses were mainly, unspecified febrile illness 44 (38.3%), malaria 40 (34.8%), and typhoid 8 (6.9%). Only a few 23 (12.8%) of the mothers had chronic medical conditions during pregnancy, such as hypertension 11 (47.8%), HIV 3 (13.0%), and diabetes 2 (8.7%). Tetanus toxoid (TT) was the commonest immunization given during antenatal 143 (98.6%). There was only a single documentation of rubella and hepatitis B vaccines. Majority 120 (90.2%) of the mothers at first trimester, 141 (85.9%) second trimester, and 156 (95.1%) third trimester were prescribed routine prenatal multivitamin. Most of the documented birth abnormalities were Down's syndrome 34 (15.2%); congenital hydrocephalus 32 (14.3%); acyanotic congenital heart defect (ACHD) 30 (13.4%); deformity of the digits 26 (11.6%); and ventricular septal defect (VSD) 20 (8.9%). See Table 5.

**Table 4:** Antenatal history

Variable	Frequency	Percentage
Where ANC was received		
Maternity home	1	0.6
General hospital	50	27.8
Private hospital	90	50.0
Health center	6	3.3
Nurses shop	3	1.7
Traditional birth attendant	5	2.8
Church	1	0.6
Not specified	24	13.3
Total	180	100.0
Past history of congenital anomaly		
Yes	2	1.1
No	152	84.4
Not specified	26	14.4
Total	180	100.0
Congenital anomaly/adverse pregnancy outcomes in siblings		
Macrosomia	1	0.6
Birth asphyxiation	1	0.6
Foot malformation	1	0.6
Not specified	177	98.3
Total	180	100.0
Family history of congenital anomaly		
Yes	1	0.6
No	152	84.4
Not specified	27	15.0
Total	180	100.0
Use of traditional birth attendant		
Yes	9	5.0
No	143	79.4
Not specified	28	15.6
Total	180	100.0
Use of herbs in pregnancy		
Yes	9	5.0
No	148	82.2
Not specified	23	12.8
Total	180	100.0
Herbal medicine used		
Herbal concoction	4	2.2
“Agbo” (local herbal concoction)	2	1.1
Unripe pawpaw water	1	0.6
Not specified	173	96.1
Total	180	100.0
Self‐medicated medicine		
Antibiotics	1	33.3
Oral contraceptives	1	33.3
Indiscriminate drug use during pregnancy	1	33.3
Total	3	100.0
Self‐medication status		
Yes	3	1.7
No	146	81.1
Not specified	31	17.2
Total	180	100.0
Acute illness in pregnancy		
Malaria	40	34.8
Febrile illness	44	38.3
Hospitalization (for unknown condition)	9	7.8
Typhoid	8	6.9
Gastro esophageal reflux disease	2	1.6
Prolonged labor	4	3.5
Rashes	4	3.5
Vaginal candidiasis	4	3.5
Total	115	100.0
Medical condition in pregnancy		
Asthma	1	4.3
Diabetes	4	17.4
Fibroid	1	4.3
Hypertension in pregnancy	13	56.5
Eclampsia	1	4.3
HIV	3	13.0
Total	23	100.0
Immunization (before/during) pregnancy		
Tetanus toxoid	143	98.6
Hepatitis B virus infection	1	0.7
Rubella vaccine	1	0.7
Total	145	100.0
Antenatal screening status		
Syphilis	57	64.8
Diabetes	30	34.1
HIV	1	1.1
Total	88	100.0
Prescriptions in first trimester		
Routine hematinics	120	90.2
Antiretroviral drugs	2	1.5
Antimalarials	8	6.0
Aldomet^®^ (methyldopa)	1	0.8
Lexotan^®^ (bromazepam)	1	0.8
Antibiotics	1	0.8
Total	133	100.0
Prescriptions in second trimester		
Routine hematinics	141	85.9
Antiretroviral drugs	2	1.2
Antimalarials	18	10.9
Aldomet^®^ (methyldopa)	1	0.6
Lexotan^®^ (bromazepam)	1	0.6
Cough syrup	1	0.6
Total	164	100.0
Reactions in third trimester		
Routine hematinics	156	95.1
Antiretroviral drugs	2	1.2
Antimalarials	2	1.2
Aldomet^®^ (methyldopa)	1	0.6
Lexotan^®^ (bromazepam)	1	0.6
Antibiotics	1	0.6
Flagyl	1	0.6
Total	164	100.0

ANC, antenatal care.

**Table 5:** Types of birth abnormality

Variable	Frequency	Percentage
Adverse pregnancy outcomes		
Acyanotic congenital heart defect	30	13.4
Down's syndrome	55	24.5
Ventricular septal defect (VSD)	20	8.9
Low birth weight	6	2.7
Congenital heart defect	10	4.5
Atrio‐VSD	5	2.2
Meningomyelocoele	11	4.9
Spinal bifida	9	4.0
Hydrocephalus (congenital hydrocephalus)	32	14.3
Omphalocele	9	3.9
Digit deformity	26	11.6
Limb malformation	3	1.3
Craniofacial/orofacial dysmorphism	5	2.2
Umblical hernia	2	0.8
Undescended testes	1	0.4
Total	224	100.0

## 4 DISCUSSIONS

Previous studies have reported that certain prescription drugs included in the case notes of the studied subjects, such as anti‐epileptics, anticonvulsants,^24^, ^25^ and antihypertensive drugs are implicated in cardiovascular malformations.^26^ However, in the present study, there were incomplete documentations regarding antenatal history, hence a causal relationship between maternal medications or environmental exposures at gestation and the documented birth abnormalities could not be fully established.

We observed that nearly all the mothers, all through the period of antenatal were prescribed routine prenatal multivitamin product containing folic acid 0.4 mg, elemental iron 17 mg, Vitamins B1 3 mg, B2 2 mg, B6 10 mg, B12 6 μg, Niacin 20 mg, Vitamin C 70 mg, Vitamin D 400 IU, Vitamin E 4 mg, Zinc 15 mg, Copper 1000 μg, Selenium 30 μg, Magnesium 150 mg, Vitamin K 70 μg, Iodine 140 μg, Natural mixed carotenoids 2 mg, Biotin 150 μg, and Pantothenic acid 6 mg. A systematic review of Cochrane database reported that prenatal supplementation with iron or iron plus folic acid given daily or weekly is effective in preventing anemia and iron deficiency at term, but there was no significant association between prenatal iron and folic acid supplementation with reduction in substantive maternal and neonatal adverse clinical outcomes.^27^ However, the WHO recommends daily oral iron and folic acid supplementation with 30‐60 mg of elemental iron and 0.4 mg folic acid for pregnant women to prevent maternal anemia, puerperal sepsis, low birth weight, and preterm birth.^1^, ^2^ Although, in this present study, the prescribed prenatal multivitamin complied with the WHO recommendation of 0.4 mg daily folic acid in pregnancy but the iron content (17 mg) is far less than the daily requirement in pregnancy. The impact of low iron intake during pregnancy and poor compliance to the antenatal medications may be connected with the documented birth abnormalities. In the present study, the level of maternal compliance to the prescribed prenatal multivitamins was not fully captured, but recent data from Ontario, Canada revealed that compliance is less than optimal among women using prenatal vitamins; hence 40% of women of reproductive age do not achieve therapeutic systemic levels of folate needed to prevent neural tube defects.^28^

In the scientific literatures, there appears to be some conflicting information regarding the safety of multivitamin use in pregnancy. For instance, one study reports that maternal periconceptional intake of vitamin E is associated with congenital heart defects (CHDs),^29^ while another study reports that Vitamin E intake during pregnancy does not carry any risk of birth abnormalities.^30^ Also, it has been reported that women with excessive serum copper concentrations have a significantly increased risk of having offspring with a CHD, whereas, a low maternal zinc status might have a correlation with CHD.^31^ In a similar report, it was stated that within the normal range of maternal serum zinc and copper concentrations, there is no variation in risk of neural tube defects but women with very high serum zinc levels may have an increased risk of neural tube defects.^32^ Further, a case‐control study provides evidence that suggests an association between concentrations of maternal zinc and the risk of orofacial clefts in offspring.^33^ Also, studies have shown that elevated placental concentrations of manganese may be associated with increased risks of neural tube defect.^34^

In the present study, unspecified febrile conditions, hypertension, and pregestational diabetes, were the most documented maternal pathologies. However, a causal relationship between these pathologic conditions and the documented birth abnormalities could not be directly established because of possible effects of the medications used in managing these disease states. However, in a population‐based retrospective cohort, febrile illness with no multivitamin use was associated with generally increased risk of some birth defects including orofacial, central nervous system, cardiovascular, limb, and abdominal abnormalities.^12^ It is recommended that women who experienced fever of 38.9°C or higher for extended period of time in the first month of pregnancy should be considered at increased risk for neural tube defects and should be provided appropriate counseling.^15^ It is on record that physiological changes early in pregnancy that manifest in gestational hypertension and pre‐eclampsia may play a role in the etiology of major birth defects, including CHD and hypospadias.^35^ In the same light, poorly controlled pregestational diabetes has also been implicated in the development of birth defects.^13^

Our study has potential limitations. Aside from the incomplete documentations on maternal medication, this work is an audit of medical records; the documented congenital abnormalities were based on the clinician's assessments. This may be limited by the clinician's level of experience in managing birth defects. However, most of the assessments were supported by adequate laboratory and other clinical investigations including X‐rays, ECG, ultrasound scan, CT scan, appropriate blood test, amongst others. Finally, it suffices to mention that our current findings contribute to the existing body of literature evidence that certain maternal pathologies may contribute to the development of birth abnormalities.

## 5 CONCLUSION

In conclusion, this retrospective audit of medical records of newborns with congenital abnormalities at the LUTH reports prevalence of maternal acute and chronic illnesses such as malaria, typhoid, hypertension, and gestational diabetes, suggesting that these pathologies or their drug treatment during pregnancy may be implicated in the development of birth abnormalities. We therefore recommend that the aforementioned pathologic states and their drug treatment during pregnancy be further investigated in future case‐control studies to fully understand their involvement in the development of birth abnormalities.

## DISCLOSURES

None declared.
